# Oncogenic Mutations Differentially Affect Bax Monomer, Dimer, and Oligomeric Pore Formation in the Membrane

**DOI:** 10.1038/srep33340

**Published:** 2016-09-15

**Authors:** Mingzhen Zhang, Jie Zheng, Ruth Nussinov, Buyong Ma

**Affiliations:** 1 Department of Chemical & Biomolecular Engineering, the University of Akron, Akron, Ohio 44325; 2 Basic Science Program, Leidos Biomedical Research, Inc. Cancer and Inflammation Program, National Cancer Institute, Frederick, MD 21702, USA; 3 Sackler Inst. of Molecular Medicine, Department of Human Genetics and Molecular Medicine, Sackler School of Medicine, Tel Aviv University, Tel Aviv 69978, Israel

## Abstract

Dysfunction of Bax, a pro-apoptotic regulator of cellular metabolism is implicated in neurodegenerative diseases and cancer. We have constructed the first atomistic models of the Bax oligomeric pore consisting with experimental residue-residue distances. The models are stable, capturing well double electron-electron resonance (DEER) spectroscopy measurements and provide structural details in line with the DEER data. Comparison with the latest experimental results revealed that our models agree well with both Bax and Bak pores, pointed to a converged structural arrangement for Bax and Bak pore formation. Using multi-scale molecular dynamics simulations, we probed mutational effects on Bax transformation from monomer → dimer → membrane pore formation at atomic resolution. We observe that two cancer-related mutations, G40E and S118I, allosterically destabilize the monomer and stabilize an off-pathway swapped dimer, preventing productive pore formation. This observation suggests a mechanism whereby the mutations may work mainly by over-stabilizing the monomer → dimer transformation toward an unproductive off-pathway swapped-dimer state. Our observations point to misfolded Bax states, shedding light on the molecular mechanism of Bax mutation-elicited cancer. Most importantly, the structure of the Bax pore facilitates future study of releases cytochrome C in atomic detail.

B-cell lymphoma-2-associated X (Bax), the apoptosis regulator belonging to the B-cell lymphoma-2 (Bcl-2) protein family, seals a cell death warrant by promoting elimination of infected or damaged cells via a structural transition pathway of monomer → dimer → membrane pore formation^1^,^2^,^3^. In healthy mammalian cells, the inactive Bax proteins mainly exist in the cytosol, and occasionally also on the mitochondrial outer membranes in the monomeric state^4^,^5^,^6^. External or internal apoptotic signals trigger a series of the Bax structural changes, resulting in either heterodimerization with other pro-survival Bcl-2 proteins or homodimerization^7^,^8^,^9^,^10^. Increasing evidence suggests that Bax-induced cell death is eventually related to its interactions with the mitochondrial outer membrane (MOM)^11^,^12^,^13^,^14^,^15^,^16^,^17^. Different molecular mechanisms were proposed to clarify the MOM-involved pathways for Bax-elicited cell death, including opening of the mitochondrial voltage-dependent anion channel (VDAC)^18^,^19^, forming mitochondrial oligomeric pores (MAP)^20^, and activating the mitochondrial apoptosis-induced channel (MAC)^21^,^22^. The formations of the Bax mitochondrial oligomer pores (MAP) from the Bax homodimers may releases cytochrome C and other apoptosis-induced factors (AIFs) from the mitochondrial intermembrane space to the cytoplasm, which provokes the proteolytic cascade that eventually ensures the cell’s demise^2^,^23^,^24^,^25^. Various experiments have been designed to characterize morphologies of MAP structure and reveal molecular mechanisms of the MAP formation^26^,^27^,^28^. These studies provided important information about supramolecular organization of Bax during apoptosis. However, as the ultimate point of functional pathway, the atomic structure of the Bax oligomeric pore at the mitochondria is still undetermined to date, preventing the atomic understanding of the Bax functionalities, although recent double electron-electron resonance (DEER) spectroscopy measurements provided information related to residues which are in contact in Bak and Bax^29^,^30^.

The structures of Bax proteins, both monomers and dimers, have been well characterized by NMR, cryo-EM, and X-ray^7^,^31^,^32^. Monomeric Bax has a globular shape consisting of bundle of nine α-helices connected by ten flexible loops^7^. As typical anti-survival proteins, Bax contains three highly conserved Bcl-2 homology (BH) motifs, BH1 (α4-α5), BH2 (α7-α8), BH3 (α2-α3) and BH4 (α1), respectively^33^,^34^. It has been well known that different domains of Bax proteins are in charge of different cellular activities, i.e., BH3 is involved in the hetero-association with the pro-survival counterparts, while the C-terminal segment (α9) is responsible for the insertion of the MOM into the cell^3^,^7^. Before activation to form large pores at MOM^17^, Bax first forms dimers either in solution^35^ or in the membrane^29^,^36^. X-ray crystal structures of Bax proteins reveal two possible dimer conformations, known as the Core/Latch swapped (PDB code: 4BD8) and BH3-in-groove (4BDU) dimers, respectively^31^. In the swapped dimer, the Bax protein is divided into two different parts, the latch domain constituting the swapped helices (α6-α8) and the core regions consisting of α1-α5, bridged by the antiparallel extended α5-α6 helices. The BH3-in-groove dimer is two-layered, with α2-α3 and α4-α5 comprising the upper hydrophilic and lower hydrophobic surfaces, respectively. These two dimers may relate to the Bax protein pathways. The swapped dimer, whose formation has to be activated by the BidBH3 and BimBH3 peptides, is unlikely to be an “intermediate” for the Bax-induced apoptosis since its structure lacks interfaces identical to those reported in Bax oligomers during apoptosis^31^,^37^. Thus, it has been suggested that the swapped dimer is the off-pathway species towards the Bax-mediated apoptosis^31^. By contrast, the BH3-in-groove dimer, exhibiting similar residue-residue distances in the MAPs, is believed to be on-pathway, and recent data suggest that it acts as the structural unit of the oligomeric pores^29^,^30^,^35^,^36^. Thus, another important question related to the Bax structural biology is how to correlate the multiple dimer conformations with MAP formation and possible cancer implication.

While somatic mutations of the BAX and BAK genes are rare in common cancers^38^, sixteen missense mutations have been documented in the Catalogue of Somatic Mutations in Cancers (COSMIC) database^39^. However, the underlying mechanisms remain elusive. Since the functional mechanism of Bax involves a structural change from monomer to dimer and oligomeric pore formation, it is natural to ask whether these cancer-related mutations may affect its structural changes and thus its oligomerization pathway, which in turn would alter its biological functions.

In this study, we computationally examined two crucial problems: (1) the Bax oligomeric pore structure in atomic details, (2) possible molecular mechanisms of Bax cancer mutations related to Bax dimerization and oligomerization pathway. For the first time, we provided the complete atomic structure of Bax oligomeric pores at the membranes, which were able to reproduce the experimentally observed residue-residue contact distributions. With this model, we systemically investigated the mutation effects on the Bax monomer, swapped dimer and oligomeric pore structures using all-atom and coarse-grained molecular dynamics simulations. We selected two point mutations in Bax proteins (S118I in lung cancer and G40E in colon cancer) from the COSMIC database to study the mutation effects on the Bax structural transition pathways. In a recent study of oncogenic mutation effects on apoptotic network, it was found that S118I belongs to a group that shifts the apoptosis to a higher death threshold^40^. Our all-atom explicit-solvent simulations showed that G40E and S118I render the Bax monomer more flexible and over-stabilize the swapped Bax dimer. Our energy calculations have shown that the apo form of the off-pathway swapped dimers is thermodynamically less stable than the monomer. However, both G40E and S118I mutations greatly lowered the dimerization energies of the Bax swapped dimer and thus could promote the off-pathway dimerization. We further found that the mutations only slightly destabilize the oligomeric pore, suggesting the major consequence of the mutations being the off-pathway shift. The models and the simulations provide the structural, dynamic and energetic properties of Bax, which provide insight into Bax proteins and their potential cancer-causing mechanisms^41^. Our study suggests that by destabilizing the Bax monomer and over-stabilizing the Core/Latch swapped dimer, cancer mutations could trap the Bax protein deflecting it away from the apoptosis pathways.

## Results

### Mutations Destabilize the Bax Monomer

**Figure 1:**
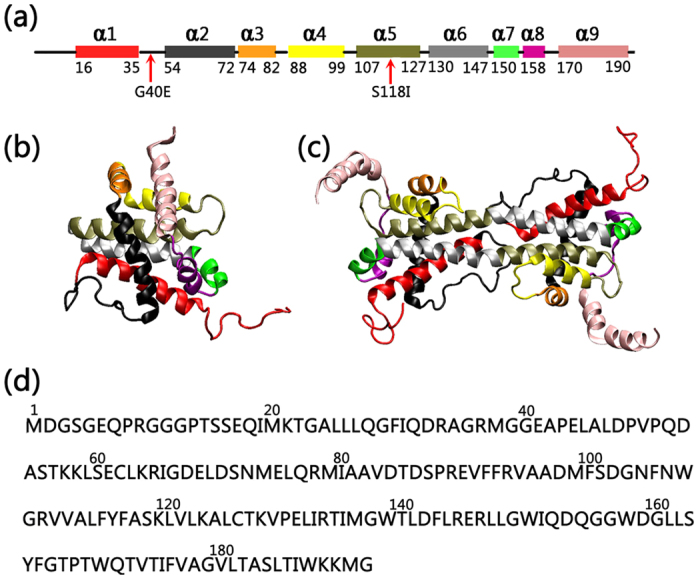
(**a**) The locations of mutations are mapped on a schematic representation of the protein secondary structure. (**b**) The structures of the Bax monomer (lift) and dimer (right), and (**c**) the sequence of the Bax protein. α helices in Bax protein are represented by different colors. Color code for the helices: red (α1), black (α2), orange (α3), yellow (α4), tan (α5), silver (α6), green (α7), purple (α8) and pink (α9).

The Bax monomer consists of nine α helix segments connected by ten flexible loop regions (Fig. 1b). The α helices in the Bax monomer intertwine to form a globular structure, with one loop region (residue 1–16 referred as loop1) floating outside just like “antenna”. The α helices show a higher structural stability relative to the loop regions. In both wild-type and mutated Bax, the most flexible regions are identified as the terminal loops (loop1 and loop10), which exhibit RMSF values up to 9.4 Ǻ (Fig. S1). Visual inspection of the trajectories also confirms that the overall structural fluctuations for monomeric Bax proteins mainly derive from continuous swings of these two terminal fragments, while the other parts of the protein remain compact throughout the simulations.

**Figure 2:**
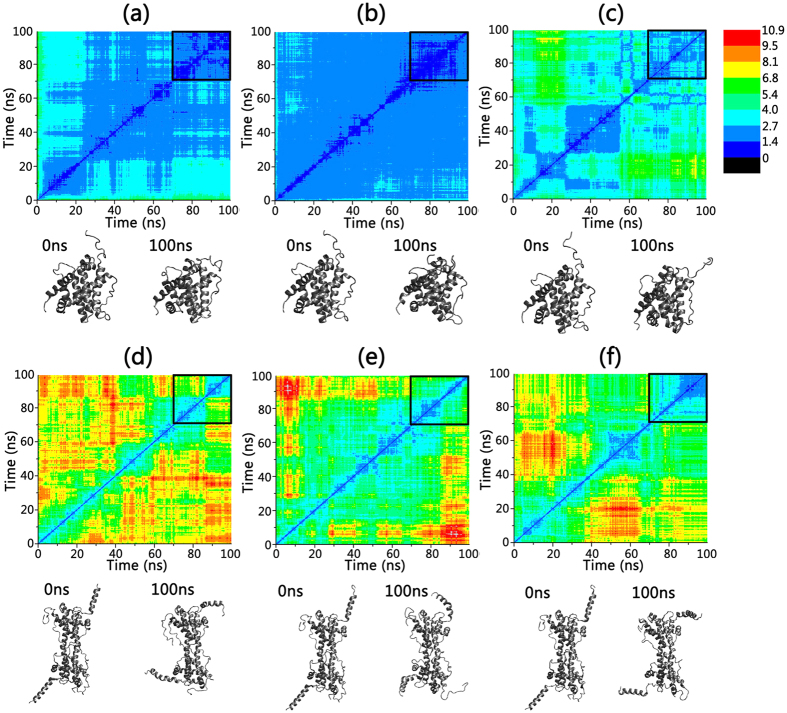
2D backbone RMSD matrix plots as function of simulation time and the initial and final structure snapshots for (**a**) wild-type Bax monomer, (**b**) Bax G40E monomer mutant, (**c**) Bax S118I monomer mutant, (**d**) wild-type Bax dimer, (**e**) Bax G40E dimer mutant, and (**f**) Bax S118I dimer mutant.

**Figure 3:**
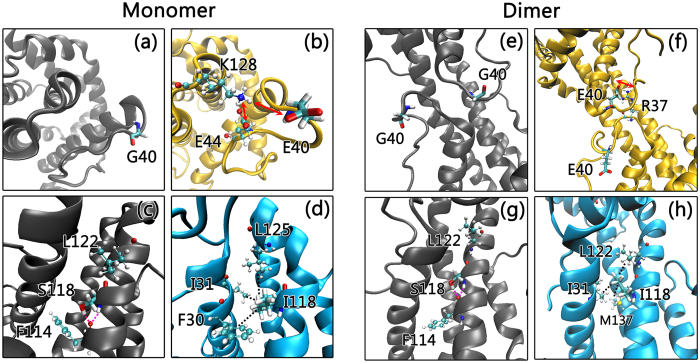
Close-up of mutation sites for (**a,c**) wild-type Bax monomer, (**b**) Bax G40E monomer mutant, (**d**) Bax S118I monomer mutant, (**e,g**) wild-type Bax dimer and (**f)** Bax G40E dimer mutant, and (**h**) Bax S118I dimer mutant. The secondary structures of Bax protein are represented in NewCartoon. Wild-type Bax proteins, G40E mutants and S118I mutants are colored in gray, yellow and cyan, respectively.

Figure 2a–c depict the two dimensional root-mean-square deviation (2D-RMSD) plots for the wild-type Bax monomer and its G40E and S118I mutants, in which each color point represents the RMSD between the frame conformation on the x-axis and on the y-axis. RMSD values are colored from blue to red covering the range from 0 to 10.9 Ǻ. After the initial structural relaxation, the 2D-RMSD values of the wild-type Bax monomer and the two mutants go down gradually and become steady at ~70 ns, suggesting that the monomer systems achieved equilibrium in the last 30 ns of the simulations. Comparisons of the 2D-RMSD values in the last 30 ns trajectories between the wild-type and mutated Bax monomers, combined with visual inspection, clearly reveal that the two mutations destabilize the Bax monomers to different extents. In wild-type Bax, G^40^, an amino acid which lacks a sidechain in the loop2 region, interacts weakly with neighboring residues and is consistently exposed to water (Fig. 3a). When a small residue (G^40^) is mutated to polar/charged amino acid (E^40^), the negative charge engages with the positively-charged amino acid K^128^ to form a salt bridge (Fig. 3b). However, the salt bridge of E^40^-K^128^ is dynamic, because E^40^ and E^44^ competitively form a salt bridge with K^128^. The E^40^-K^128^ salt bridge imposes strong attraction on the α1 helix region, leading to its twist and fissure. The competition between the α1 helix twisting with disruption of E^44^-K^128^ salt bridge and formation of the E^40^-K^128^ salt bridge give rise to the pronounced structural fluctuation around the mutation site. Although the 2D-RMSD value for G40E (1.4 ± 0.2 Å) is close to the wide-type (1.5 ± 0.3 Å), the root mean square fluctuation (RMSF) values for α1-α2 regions of G40E mutants (~0.94 Ǻ) are much higher than those of the wild-type Bax monomer (~0.68 Ǻ), suggesting an increased residue fluctuations at the mutation site (Fig. S1). The local structural changes by the G40E mutation also lead to the reduction in the number of intramolecular hydrogen bonds in the G40E mutant (~176 hydrogen bonds), as compared to the original 188 hydrogen bonds in wild type Bax (Table 1). Consequently, the loss of these nonbonded contacts in the G40E mutant leads to slightly higher potential energy relative to the wild type (Table S1).

Similar to the G40E, the S118I mutation also induced the destabilization of the Bax monomer but to a greater extent. The 2D-RMSD values of Bax S118I mutant in equilibrium (2.5 ± 0.7 Ǻ) are much higher than those of the wild-type monomer (1.5 ± 0.3 Ǻ) in Fig. 2a,c. The S118I mutation, from hydrophilic to hydrophobic residue, causes large changes in residue-residue contacts and long-range non-bonded associations. In wild-type Bax, S^118^ forms two typical backbone-backbone hydrogen bonds with its neighboring residues F^114^ and L^122^ in α5. The side chain of S^118^ also interacts with the backbone of F^114^ to produce dual hydrogen bonds (Fig. 3c). This clearly suggests that residue 118 is involved in intra-helix associations in the wild-type Bax monomer. When S^118^ is mutated to I^118^, residue 118 presents several inter-helix contacts (Fig. 3d). I^118^ obtains side-chain contacts with L^125^, I^31^, and F^30^. Interestingly, binding between I^118^ and these hydrophobic residues induces twisting α5, which implies the collapse of the original α-helix packing upon S118I mutation. This makes the S118I mutant lose 15 hydrogen bonds and 1 salt bridge (Table 1), giving rise to an increase of the overall potential energy (Table S1).

### Mutants Over-stabilize the Core/Latch Swapped Dimer

Bax dimerization is a typical symptom of programmed cell death^8^,^42^. Bax swapped dimer is composed of two Bax monomers associated in an anti-parallel manner with the α9, loop 10 and loop 1 regions exposed to bulk phase (Fig. 1c). The exposed segments exhibit much higher structural flexibility and larger RMSF values of up to 21.4 Ǻ (Fig. S1). Similar to the Bax monomer, the α helix regions of the dimer are generally more stable than the adjacent loop segments. The structural properties of two monomers in the dimer exhibit good symmetry, as evidenced by the fact that the residue-based RMSF profiles for the two chains in the dimer fit well each other (Fig. S1).

Different from the Bax monomers, the mutations stabilize the structure of the Bax swapped dimer. As indicated in Fig. 2d–f, the 2D-RMSD values for the G49E (4.2 ± 1.4 Ǻ) and S118I (3.3 ± 1.2 Ǻ) mutants in equilibrium are much lower and steadier than in the wide-type Bax swapped dimer (4.8 ± 1.8 Ǻ), suggesting that the mutants enhance the overall structural stabilities. This is also supported by the strengthened intramolecular interactions in the two mutants. In the wild-type Bax dimer, G40 has negligible residue-residue contact, floating solely in the bulk phase (Fig. 3e). When G40 is mutated to E40, the loop 2 region is greatly stabilized by the newly-formed inter-chain E^40^-R^37^ salt bridge (Fig. 3f). Furthermore, the intramolecular hydrogen bonds in the Bax G40E dimer mutant are greatly strengthened to 356, further confirming that the G40E mutation stabilizes the overall structure of Bax swapped dimer. The residue contacts for S^118^ in wild-type Bax swapped dimer are quite similar to those in the Bax monomer (Fig. 3g). The introduction of the S118I mutation enhances the hydrophobic interactions between S^118^ and L^122^, I^31^, M^137^ in the swapped dimer (Fig. 3h). As compared to the wild-type swapped dimer, the number of hydrogen bonds and salt bridges in the S118I dimer mutant are increased by 28 and 5, respectively, leading to a more favorable potential energy.

In order to examine the effect of the mutations on the dimer stability, we calculated the dimerization energies of the Bax protein from the averaged structures in the last 10 ns of the trajectories using the generalized Born molecular volume (GBMV) algorithm. The GBMV implicit solvent model with the CHARMM force field estimates the dimerization energy by calculating the energy differences between monomeric and dimeric states of Bax^43^. Each structure was subject to the 400 steps minimizations to eliminate the structural fluctuations in the MD simulations. The dimerization energies were calculated by ΔE = E_dimer_ - 2*E_monomer_, where ΔE denotes the dimerization energy, E_dimer_ denotes the GBMV energy for the E_dimer_ and E_monomer_ is the GBMV energy for the monomer. As shown in Table 2, the dimerization energy cost of the wild type Bax experiences a rise of 41.8 kcal/mol. Such a high repulsive energy clearly suggests that formation of swapped dimer by wide type Bax is not a spontaneous process. This is in good agreement with experiments that swapped dimerization of Bax peptides have to be triggered by octylmaltoside or BimBH3 with CHAPS. Interestingly, the dimerization energy changed to −36.0 kcal/mol for the G40E mutant. This phenomenon became much more pronounced for the S118I mutation, which exhibits dimerization energy of −133.1 kcal/mol. Such a trend is reproduced using MMPBSA calculations as well. The change of repulsive to attractive dimerization energy suggests that two single residue mutations could dramatically promote a swapped organization. It is well established that the swapped dimer is off-pathway for the Bax-induced cell death cascade^31^. The two cancer-involved mutations greatly reduced the energies for the Bax off-pathway dimerization, which would impede cell death by preventing the Bax proteins from following normal programmed cell death.

### Atomic Structural Models of Stable Bax Oligomeric Membrane Pores

**Figure 4:**
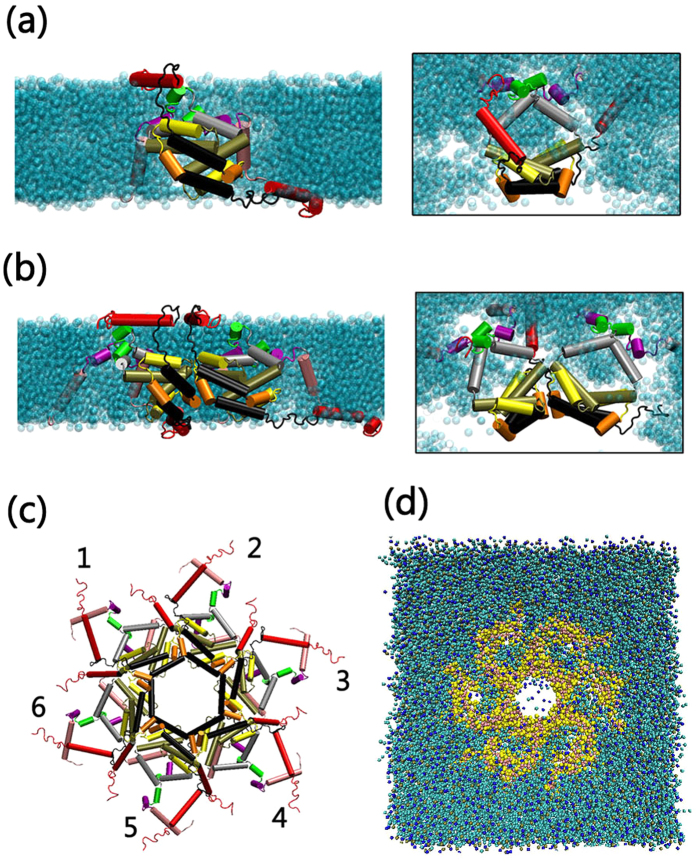
Schematic side and top views of the structural arrangements for (**a**) Bax dimer and (**b**) dimer-dimer interfaces in the membranes. (**c**) A schematic representation of the all-atom structure and the (**d**) coarse-grained model of thesix-dimer Bax oligomeric pore. The secondary structures of Bax in **(a–c**) were represented as cartoons with the color codes same as the Fig. 1.

Bax and Bak oligomeric membrane pores are the direct causative agents for cell death by permeating mitochondrial membranes^35^,^36^. Under specific conditions, Bax and Bak can individually aggregate to form oligomeric membrane pores, triggering programmed cell death by permeating mitochondrial membranes and releasing the toxic factors from the mitochondria to the cytoplasm^42^. Bax and Bak are highly homologous with similar sequences, secondary structures and biological functions^3^,^30^. Aluvila *et al*.^30^ performed DEER and EPR experiments to clarify the morphology of the Bak oligomeric pores by measuring the intra- and inter-peptide residue-residue distances. They proposed that in lipid bilayers, Bak dimers can associate to form MOM pores with a dimeric structure identical to that of the Bax BH3-in-groove dimer, and that these dimers have α3:α3’ and α5:α5’ oligomerization interfaces. Parallel works conducted by Bleicken *et al*.^29^ directly investigated the active Bax oligomeric pores in the mitochondria, measuring the residue-residue distances and further confirming that Bax oligomeric pores in the mitochondria are dimer-dimer aggregates with the typical BH3-in-groove dimeric structures. The remarkable structural similarity between Bax and Bak proteins clearly suggests that they may share similar membrane aggregation behavior with similar membrane pore architectures. In the current work, we firstly modeled the atomic structure of the Bax oligomeric pore using in-house modeling codes, based on the structural information of both Bak and Bax oligomeric pores in the membranes (details can be found in Method and Materials). The size and molecular weight of the Bax oligomeric pore are still debated. Bax oligomeric pores may have tunable sizes and molecular weights^28^,^42^. However, as suggested earlier, the Bax and Bak oligomeric pores, regardless of their sizes and molecular weights, are assemblies of dimers, sharing a common pore architecture with similar intra- and inter-dimer interfaces^28^,^29^,^30^. Thus, we only selected and modeled the six-dimer Bax oligomeric pore as a representative pore structure to study their structural and dynamic properties. Figure 4 presents the atomic structures for the constructed Bax oligomeric pores.

**Figure 5:**
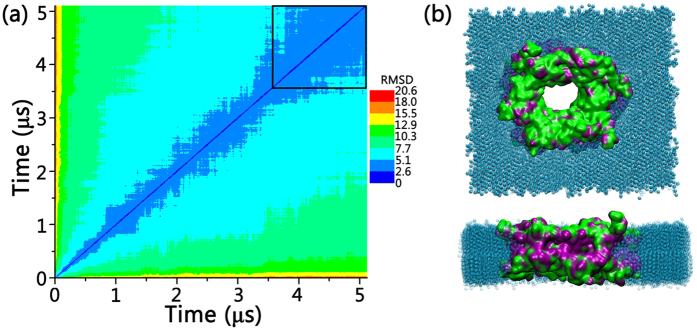
(**a**) 2D backbone RMSD matrix plots for wild-type Bax oligomeric pore, and (**b**) top and side views of the equilibrium Bax six-dimer oligomeric pore. The Bax oligomeric pore in (**b**) is represented in QuickSurf with the exposed hydrophilic residues colored in green and hydrophobic residues colored in purple. The coarse-grained lipids are shown in VDW in cyan.

The modeled Bax membrane pore system was run for 5.0 μs. As indicated by the 2D-RMSD profiles in Fig. 5a, Bax oligomeric pore experienced initial structural relaxation and decayed subsequently. In the last 1.5 μs, the 2D-RMSD values reduced to ~3.5 Ǻ and became stable, indicating that the Bax oligomeric pore in the simulations successfully achieved the equilibrium state. The Bax oligomeric pore preserves the original structural integrity and exhibits reasonable stability throughout the trajectory. All the inter- and intra-dimer interfaces were well maintained. Residues in the oligomeric pore generally have weak fluctuations with RMSF values of 1.0–4.5 Ǻ, except for several water-exposed surface residues with higher RSMF (5.0–14.5 Ǻ). We also monitored the distributions of the hydrophilic and hydrophobic residues at different surfaces of the Bax pore (Fig. 5b). It can be seen that the parts of the pores facing the membranes are mainly hydrophobic residues, while the residues in the inner cavity and at pore surfaces are hydrophilic ones. Such residue distribution in Bax oligomeric pore is expected to stabilize the overall pore structures in lipid membranes. Combined with the good stability of the systems, these results are in line with the structural rationale and reliability of our Bax pore model.

### Intra- and Inter-residue Distances in Modeled and Experimentally-detected Bax Membrane Pores

**Figure 6:**
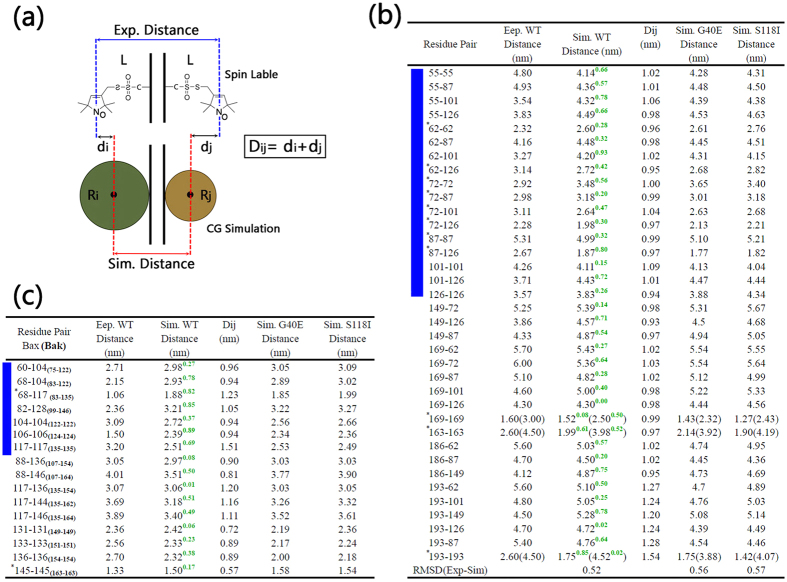
(**a**) Schematic diagram for the critical values (*D*_*ij*_) in the calculation of the discrepancy between experimental and simulated residue-residue distances and (**b,c**) the experimental and simulated residue-residue distances for the wide-type, G40E mutated and S118I mutated Bax oligomeric pores. Experimental data in (**b**) and (**c**) are obtained from Aluvila’s^30^ and Bleicken’s^29^ work, respectively. In (**b,c**), WT denotes the wild-type Bax oligomeric pore, and the green numbers at the top right are the differences between experimental and simulated residue-residue distances. The residue pairs with the bimodal distance distribution are marked by *, and all residue pairs in α2-α5 BH3-in-groove domain are highlighted by left blue stripes. The details of the calculations of the residue-residue distances can be found in Methods and Materials.

To further test the validity of our models, the residue-residue distances in the theoretical Bax oligomeric pore were measured and compared with experimental data. Due to the lack of atomic details in coarse-grained models, the residue-residue distances in the simulations have to be defined as the distances between the centers of mass of the two coarse-grained residue clusters, while that in the DEER and EPR experiments were measured between two nitroxide spin labels at the amino acid side-chains (Fig. 6a). The differences in the measuring methods induce intrinsic variances between the simulated and experimental residue distances. In order to quantify the discrepancy, for a given residue pair (i,j), we first measured the radius of coarse-grained clusters in the simulations, denoted as Ri and Rj, and the length of nitroxide spin labeled residues denoted as L. Then, the critical values for residue-residue distance differences (*Dij*) between the simulations and experiments were calculated by *Dij* = *di* + *dj*, where *di* = *L-Ri* and *dj* = *L-Rj*. *Dij* values for all residue pairs were listed in Fig. 6b,c, in which it can be seen that generally the ~10 Ǻ deviations are reasonable. Comparing with experimental results, our simulated models deviate from experimental values by far less than the 10 Ǻ mark. In the experimental DEER study of Bax in the membrane via the multilateration method^32^, the RMSD (for measured distances) for monomer is around 4.9 Ǻ and 7.8 Ǻ for dimer. The absolute deviation for the spin-labeled pair 55–101 is around 9.8 Ǻ^32^. As can be seen in Fig. 6b, the RMSD between our simulation and experimental values is only 5.2 Ǻ.

Importantly, two works consistently suggest that α2-α5 segments formed dimeric structures that are structurally homologous to the “BH3-in-groove” dimer^29^,^30^. Analysis of the residue type indicates that the surfaces of the α2 and α3 largely consist of hydrophilic residues, while those in the α4 and α5 contain considerable hydrophobic residues. Combined with the proposed model by Aluvila *et al*.^30^, it can be reasonably assumed that α2-α3 are exposed to water solution in the inner cavity of the oligomeric pores, while α4-α5 face the hydrophobic core of the membranes. The proposed orientations for the α2-α5 segments were verified by distance comparisons between the simulated and experimental Bax oligomeric pore from Bleicken *et al*.^29^. In Fig. 6b, the simulated residue-residue distances in Bax pores for the 55–55, 55–87, 55–101, 62–62, 62–87, 62–101, 62–126, 72–72, 72–126, 87–87, 87–126, 101–101,101–126 and 126–126 residue pairs present excellent consistency with the experimental measurements, with the minor differences falling within the reasonable experiment-simulation derivation ranges. Visual inspection of the trajectory confirms that the overall intramolecular residue contacts and structural integrity for α2-α5 region are well preserved throughout the simulations.

Three orientations for α6-α8 segments were proposed previously, i.e., pseudo parallel and pseudo anti-parallel by Aluvila *et al*.^30^, and arms of the “clamp” by Bleicken *et al*.^42^. In this work, the anti-parallel structural organization for α6-α8 segments were employed, since its chiral architecture can facilitate the inter-chain binding and produce more potent local residue-residue associations that may stabilize the overall pore structures. In the simulations, this model gives rise to a very stable α6-α8 cluster, perfectly reproducing the experimental residue distances not only for those within α6-α8 domain but also between α6-α8 and other segments. Specifically, 163–163,169–169 186–149 pairs within α6-α8 domain fit well into the experimental data (Fig. 6b). The residue-residue distances between α6-α8 and α2-α5 domains, i.e., 149–72, 149–126, 149–87, 169–62, 169–72, 169–87, 169–101, 169–126, 186–62, 186–87, are very close to the experiments with the variations lower than 7.1 Ǻ.

Experimental evidence indicates that α9 of Bax may play a role in membrane insertion^3^. Residue distances in the experiments suggest that α9 is far away from the α2-α5 domain of the Bax pores^29^. Recent computational and experimental studies indicated the conformational heterogeneity of Bax α9 dimer for apoptotic pore formation^44^. In our model, α9 was initially inserts into the membrane hydrophobic region vertically (details can be found in Method and Materials), with evenly large separation. However, we observed the spontaneous dimer formation of α9 (Fig. S2) during our simulation. The equilibrated orientation of α9 in the simulations produces data compatible with experimental distance of residue 193–193 (Fig. 6b). The distances between two α9 segments from inter- or intra-dimers in the simulations perfectly reproduce the doublet peaks in the distance distribution profile for 193–193, further confirming the convergence of our model. The simulated mean residue distances for 193–62, 193–101,193–126, 193–87 pairs are all ~5.0 nm, consistent with the experimental values and demonstrating that the α9 segment is reasonably far away from the α2-α5 domain in our model.

Interestingly, by conducting additional comparisons as shown in Fig. 6c, we also observe that the structural arrangement in our model is also compatible with the Bak-matched Bax oligomeric pore suggested by Aluvila’s work^30^. For all α2-α5 and α6-α8, the residue-residue distances in the trajectories are consistently much lower than the maximal acceptable differences. The biggest difference between the EPR distances measured on 124–124 pair in oligomeric Bak by Aluvila *et al*.^30^, and the corresponding 106–106 pair in Bax pore. Our simulated distance is 2.39 nm, which is about 0.89 nm longer than experimental value of 1.5 nm (Fig. 6c). However, our model is more consistent with the work from by Bleicken *et al*.^32^. The experimental distance between residues 101–101 is 4.26 nm^32^, while in our model it is 4.11 nm. Recently independent work from different groups suggested that this area of Bax/Bak is in contact with the membrane^17^,^29^,^31^. Thus the longer 106–106 distance in our model is more consistent with the above work. These data suggest that our model captures well the experimental structural information from both Bax measurements and Bak-matched data, supporting it as reasonable and reliable.

### Minor Mutational Effects on the Bax Oligomeric Membrane Pores

**Figure 7:**
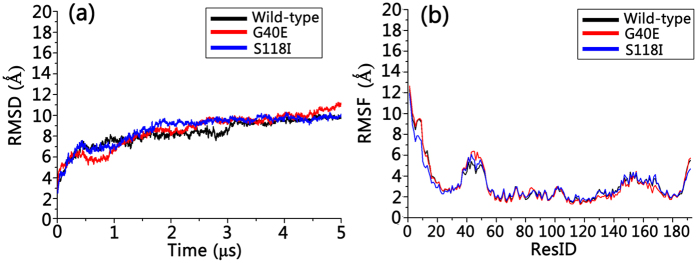
(**a**) Time-dependent backbone RMSD and (**b**) residue-based backbone RMSF profiles for wild-type, G40E mutated and S11I mutated Bax oligomeric pores.

Since different Bax oligomeric pores may have similar pore architecture and intra- and inter-chain residue contacts, we only selected the six-dimer Bax oligomeric pore as a representative pore structure to study the effects of mutations on structural changes. Simulation trajectories reveal that both G40E and S118I oligomeric pore mutants maintained well their structural integrity, with similar structural and dynamic properties as the wild type Bax pore. By measuring the residue distances as shown in Fig. 6b,c, we found that the G40E and S118I mutations only slightly increased the deviation from the experimental structure. Comparing with experimental measured residue distances, the RMSDs are 0.52 nm, 0.56 nm, and 0.57 nm for wild-type Bax, G40E mutant, and S118I mutant, respectively. For the G40E mutant, the largest deviation is the 62–101 pair (1.08 nm); and for S118I the largest deviation is the 193–193 pair (1.39 nm). Otherwise, the Bax oligomeric pores maintain their residue distribution profiles upon the two mutations, suggesting that the mutations have minor impacts on the structures of the oligomeric pores. Meanwhile, the G40E and S118I mutations do not make obvious changes in the dynamic properties of the Bax oligomeric pores either. In Fig. 7a, the time-dependent RMSD profiles of G40E and S118I overlap perfectly with that of wild-type Bax oligomeric pore, suggesting that the mutations exert almost negligible influence on the structural fluctuations of the pores. The similar residue-based RMSF profiles for mutants and wild-type Bax oligomeric pores in Fig. 7b further confirm these minor effects. The insignificant residue mutation effects on the Bax oligomeric membrane pores may be straightforwardly expected since the structural characteristics of the Bax pores are mainly determined by the dimer-dimer and dimer-membrane interactions. Structural changes induced by single residue mutations can be greatly compensated by the constraints from the membranes and neighboring dimers. Interestingly, S118I mutation seems increase interaction of α9, as can be seen by shorter inter-residue distances of residue 193 with its self and other residues (Fig. 6b,c). Taken together, these findings imply that the mutations have minor influence on the downstream structural transformation of Bax proteins.

## Discussion

Bax proteins are key regulators of programmed cell death, but high-resolution structures in lipid membranes still remain unsolved. Consequently, the mechanisms on the molecular level of their cancer-related mutations are also still a mystery. In this work we systematically modeled the monomer, dimer and mitochondrial oligomeric pores using multi-scale MD simulations and investigated the effects of cancer-related mutations on the structural and energetic properties. Our results indicate that both the G40E and S118I mutations effectively destabilize the Bax monomer by switching residue-residue contacts adjacent to the mutation sites. More interestingly, we observed that the G40E and S118I mutations greatly reduced the dimerization energies for the Bax swapped dimers, from repulsive to attractive. This change clearly suggests that these mutations turn the Bax swapped dimerization from a non-spontaneous into a spontaneous process, potentially trapping Bax in an off-pathway dimer state and interfering with Bax-induced apoptosis. We have successfully constructed atomic models of the Bax/Bak oligomeric pore using the residue-residue distances obtained from experiments. The modeled pores reproduced well the experimental data with good structural stability. Simulations of the pores formed by the G40E and S118I mutants indicated that mutational effects on the six-dimer Bax oligomeric pores were insignificant, suggesting that the cancer-related mutations mainly affect the pathway prior to the pore formation, driving it to an off-pathway misfolded dimer. As such, these results provide molecular clues for Bax-mutation-mediated cancers.

Bax is proposed to induce toroidal pores and the pore formations are sensitive to lipid composition^42^,^45^,^46^. Native mitochondrial outer membranes and protein-free liposomes display different permeabilization kinetics, and native membranes respond to lower concentrations of Bax^47^. Thus, Bax may have similar pore structures but different kinetics. In this work, we focus on the relative stabilities of Bax dimer and pore in the membranes caused by mutations, rather than the process of the Bax pore formation. Phosphatidylcholine (PC) is the most abundant lipid in mitochondria membranes, occupying over 44% weight of the total phospholipids^48^. Thus, we used DSPC as the representative lipids to mimic membrane effects. Our results in Fig. 5 show that the modeled membrane provides reasonable mixture of hydrophobic/hydrophilic surfaces to stabilize the Bax pores, indicating that the modeling of membranes in this work can reproduce well the experimental results. As shown in Figure S3, our equilibrated pore and membrane structure shows toroidal pore feature, in agreement with previous studies^42^,^45^,^46^. Overall, our studies and comparison with the latest experimental results for the Bax pore structure revealed that our modeled structures agree very well with both Bax and Bak pores, pointing to a converged mechanism for Bax and Bak pore formation.

**Figure 8:**
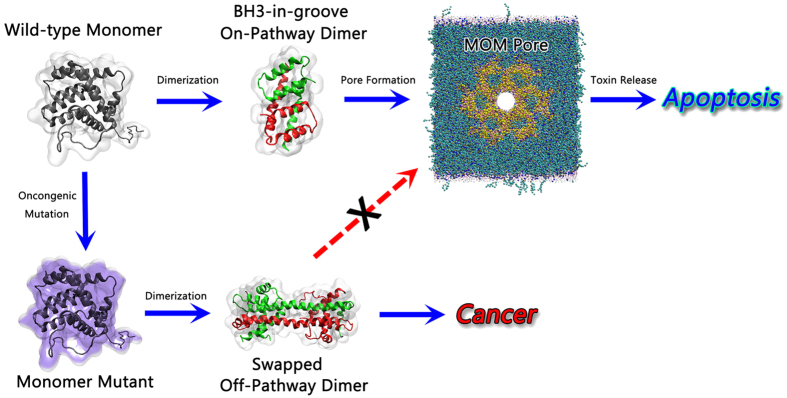
Schematic illustration of the normal (top) and oncongenic mutation allostric (bottom) pathways for Bax proteins.

Allostery is an intrinsic property of many proteins^49^,^50^,^51^,^52^. Constitutive allosteric activation by oncogenic mutations is at play in virtually all biological processes in the living cells^53^,^54^,^55^. The allosteric effect could be induced by various chemical and biological events, such as single residue somatic mutations and may result in cancer development^56^,^57^,^58^, via ligand binding that prohibit or enhance normal protein functions^59^,^60^,^61^. This work clearly suggests that the oncogenic G40E and S118I mutations interfere with the Bax functional pathways by triggering protein allostery. For wild-type Bax, the native highly populated active state in the folding free energy landscape may warrant conformational transformation of the monomer → on-pathway dimer → oligomeric pore, guaranteeing its cell apoptosis function (Fig. 8). Our simulations suggest that single cancer-triggering residue mutation can effectively destabilize the Bax monomer and stabilize the off-pathway Bax swapped dimers. Mutational events destabilize the monomers increasing their potential energy; however, they lower the potential energies of the dimers. Such changes in the energy landscape shift Bax towards the off-pathway dimer. The reversals of the state populations by the allosteric effects push the Bax monomers into the off-pathway swapped-dimer funnels in the free energy landscape. When the off-pathway Bax dimers become more populated, the normal cascade of Bax proteins will be reduced, promoting tumors by eliminating normal cell apoptosis (Fig. 8). Of note, the mechanisms of mutant-controlled energy landscape shift that can either promote or impede the swapped dimer, are not limited to Bax, but observed for other proteins, including Grb7^62^, E-cadherin^63^, p38α^64^, LRRK2^65^, Protein L^66^, SPAK^67^ and cytochrome complexes^68^. This suggests that the triggering or blocking swapped protein assemblies by mutations is a general phenomenon that plays an important role in human metabolism and broadly in the cell^69^, including in membrane pores as is the case here.

## Materials and Methods

### All atom MD simulations of full length Bax monomer and swapped dimer

Initial all-atom coordinates of the Bax monomer (PDB code: 1F16) and swapped dimer (PDB code: 4BD8) were extracted from the protein data bank^7^,^31^. As shown in Fig. 1, the missing residues in Bax swapped dimer were extracted from the Bax monomer structures, and fused into the Bax swapped dimer structures using the VMD scripts. The positions and orientations of the missing fragments were assigned by in-house Tcl scripts. The full length Bax swapped dimer was subjected to 5000 minimizations steps and a short 1 ns MD simulation, in which the missing residues were fully relaxed while the pre-existing parts of the dimer were harmoniously constrained. The N- and C- termini of the Bax swapped dimer were capped by NH3 + and COO- groups, respectively. The involved basic (His, Arg and Lys) and acidic (Asp and Glu) residues are normally charged. In order to study the mutational effects on the Bax swapped dimer, the G40 and S118 were mutated to Glu and Ile, respectively, using the VMD mutation package. Similarly, the mutations of G40E and S118I were simultaneously conducted on the Bax monomer.

### All-atom MD simulation protocols

Gromacs-4.6.5 was employed to perform individual 100 ns simulations throughout. The SPCE solvent model and CHARMM27 force field with the CAMP potential correction were applied to all systems. All wild types and mutants for Bax swapped dimers and monomers were solvated in cubic water box with the minimal margin of 15 Ǻ from any edge of the water box to any peptide atom. Na^+^ and Cl^−^ ions were added to the systems, neutralizing the charges and achieving ion strength of ~150 mM. Before the simulation, all systems were fully optimized by steepest decent minimization with the protein backbone fixed, followed by additional minimizations without any constraints. Short NVT simulations were performed on all systems to gradually heat the simulation boxes from 0° K to 310° K. Likewise, short NPT simulations were conducted to adjust the system pressures. All covalent bonds involving hydrogen atoms were constrained by the LINCE algorithm. Short-range van der Waals (VDW) interactions were calculated by the cut-off method with the potential-shift-Verlet modifier. Long-range electrostatic forces were described by the particle mesh Ewald (PME) method. A 2 fs time step was used throughout the simulations. All the analyses were conducted using the CHARMM, VMD and in-house scripts^70^.

### Bax coarse-grained mitochondrial oligomeric pores

The coordinates of BH3-in-groove Bax dimer (4BDU)^31^, as well as the Bax monomer (1F16)^7^, were used as structural templates to build the Bax oligomeric pore. Since Bax and Bak have the same homology, sharing similar secondary structures and BH3-in-groove dimer structures, the experimental residue-residue distances obtained by double electron-electron resonance (DEER) spectroscopy were employed to establish the Bax oligomeric pore^29^,^30^. The BH3-in-groove Bax dimer structure (4BDU) was used as the framework for the α2-α5 domain in a pore. The domain consists of two exposed surfaces, i.e., the α2:α3 and α4:α5 surfaces. Since the exposed areas at the α2:α3 surface largely consist of hydrophilic residues, while those for α4:α5 surfaces mainly contain hydrophobic residues, we assigned the α4:α5 surface facing the membranes and α2:α3 surface exposed to the waters in the modeled Bax pore (Fig. 4a). The initial coordinates of α1 and α6-α9 were generated from the crystal structures of the Bax monomer (1F16)^7^. According to the experimental residue-residue distances, the C-terminals of two α6 helixes in the dimer of Bax pore were stacked together with the parallel or anti-parallel arrangements^30^. We individually established these two models and found that the anti-parallel arrangement has more α7-α8 intra-dimer interactions that may stabilize the overall structure of the α6-α8 domains. The measurement of the residue distances within the established anti-parallel model for α6-α8 domain further confirmed that this model can capture well the experimental data from both Aluvila’s and Bleicken’s works^29^,^30^. Thus, the anti-parallel model of α6-α8 domain was finally selected in our models. In-house codes were used to fuse the α6-α8 domain into the α2-α5 BH3-in-groove structures. Following the experimental residue-residue distances between α2-α5 and α6-α8 domains^30^, the orientation of α6-α8 domain was fully adjusted, as shown in Fig. 4a. The α9 segment in Bax proteins has been suggested to engage in membrane insertion and be far away from the α2-α5 domain by ~5 Ǻ^3^,^29^. In order to mimic its membrane insertion state and fit into the separation distance of ~5 Ǻ from α2-α5 domain, the inclined orientation with respect to the membrane surfaces was employed for the α9 segment in our model. Experimental measurements suggested that the α1 segment in the Bax pore simultaneously separated from both α2 and α6^30^. The α1 segment does not exist either in the inner cavity or close to the core regions (α2-α5) of the oligomeric pores. Thus, the α1 region in our model was put at the hydrophilic layer of the membranes (Fig. 4a).

### Coarse-grained MD simulation protocols

The coarse-grained MD simulations were conducted using the Gromacs-4.6.5 program with the Martini force field (version 2.4). Long-range electrostatics interactions were calculated by the shift method. The temperatures (310 K) and pressure (1 atm) were controlled by the V-rescale and the Parrinello-Rahman methods with the coupling constants of 1.0 and 12.0 ps, respectively. The temperature coupling was conducted separately for the protein, membranes and ion-water atoms. Leapfrog integrator was utilized to allow an integration time step of 30 fs.

In this work, the six-dimer Bax oligomeric pore was selected to study its structural and dynamic nature. It was constructed by assembling six Bax homodimers together circularly with the α3: α3, α5: α5 dimer-dimer interfaces^30^. The established dimer-dimer interface was highlighted in Fig. 4b. It can be seen that dimers in the Bax pore mainly associated by the α3 and α5 motifs of the α2-α5 BH3-in-groove domains, while other parts generally kept separate from each other. The Bax oligomeric pore was initially established using the all-atom model, which was subject to the rounds of fine tunings (minimizations, short NPT simulations) with the CHARMM 27 force field as to relax the pore structure and eliminate the unreasonable atom overlaps (Fig. 4c). Then, the all-atom structure was mapped into the coarse-grained model using the Martini force field (version 2.4). Phosphatidylcholine (PC) is the most abundant lipid at the mitochondria membranes, occupying over 44% weight of total phospholipids^48^. Thus, the coarse-grained distearoyl-phosphatidylcholine (DSPC) lipids in Martini force field (version 2.4) were used to generate the mimicked mitochondria membranes. The established membranes and Bax pore were merged, with lipids within 3.5 Ǻ of the Bax pore deleted (Fig. 4d). Coarse-grained water beads were then employed to solvate the system with 15 Ǻ distance between the edge of box and the solvent atoms. 150 mM ion strength was mimicked by the Na^+^ and Cl^−^ ions. To study the mutation effects on the Bax oligomeric pore, G40 and S118 residues in each chain of Bax pore were mutated to E40 and I118, respectively. The mutant systems followed the exact same modeling procedure as the wile-type Bax oligomeric pore.

### Measurements of Residue-residue distances in Bax oligomeric pores

To validate our models, the residue-residue distances in Bax oligomeric pores were measured to compare with the experimental data. Two different comparisons were conducted individually with the Bax models from Bleicken’s work and Bak-mapped Bax models from Aluvila’s experiments^29^,^30^. In the simulations, the residue-residue distances were defined and measured between the center of mass of two coarse-grained residues in the last 2.0μs trajectories. The mean residue distances were obtained by normalizing all the samples. We selected different normalizing criteria in different comparisons. When comparing to Bleicken’s Bax model, we normalized the samples that had the residue distances lower than 80 Ǻ and excluded other samples, in line with the corresponding experimental protocols^29^. Of note, native Bax has 192 residues. In Bleicken’s experiments, an additional Cys193 residue was attached chemically^29^. In the distance measurements, the simulated residue 192 was selected to match the experimental Cys193. In the case of the comparisons with Aluvila’s Bak-mapped Bax model, we first matched the residues of Bax into the Bak based on their secondary structures, and normalized the samples with the residue distance lower than 50 Ǻ, consistent with the fact that signals of >50 Ǻ were not visible in the experiments^30^. For some residue pairs, the bimodal distribution profiles were observed, in which the mean distances were calculated according to the widths of individual peaks in the profiles.

## Additional Information

**How to cite this article**: Zhang, M. *et al*. Oncogenic Mutations Differentially Affect Bax Monomer, Dimer, and Oligomeric Pore formation in the Membrane. *Sci. Rep.* **6**, 33340; doi: 10.1038/srep33340 (2016).

## Supplementary Material

Supplementary Information

## Figures and Tables

**Table 1:** Intramolecular hydrogen bonds and salt bridges for both Bax monomers and swapped dimers.

	Monomer			Dimer		
	Wild-type	G40E	S118I	Wild-type	G40E	S118I
Hydrogen Bonds	188	176	173	342	356	370
Salt bridges	16	16	15	31	31	36

Intramolecular hydrogen bonds and salt bridges are calculated from the average structures of Bax monomers and dimers that are obtained from the last 10 ns trajectories. Hydrogen bonds are determined by the donor-acceptor distance and angle cutoff of 3.2 Ǻ and 60°. Salt bridges are calculated by the oxygen-nitrogen distance and 3.2 Ǻ.

**Table 2:** GBMV energies for the different systems.

	ΔE (kcal/mol)*	ΔVdW	ΔElectrostatic	ΔMM	ΔSolvation
Wild Type	41.8 (244.5 ± 28.4)	51.7	391.8	−57.6	−344.0
G40E	−36.0 (−189.2 ± 33.5)	−17.2	22.9	−20.1	−21.7
S118I	−133.06 (−193.4 ± 21.7)	−66.7	399.5	−11.2	−454.6

GBMV energies for individual system are calculated based on the averaged structures using the dielectric constant of 80 for waters and the hydrophobic solvent-accessible surface area term factor of 0.00592 kcal/mol Ǻ^2^. The dimerization energies (ΔE) were calculated E_dimer_- 2*E_monomer_, where E_monomer_ and E_dimer_ denotes the GBMV energy for Bax monomer and dimer, respectively. MM-PBSA algorithm is used to verify the GBMV results, which are calculated using the dielectric constant of 80 for waters and 4 for proteins, with the ion concentration of 0.15 M. The numbers in parenthesis are from MMPBSA calculations.
