# Supplementary material for: Oncogenic Mutations Differentially Affect Bax Monomer, Dimer, and Oligomeric Pore Formation in the Membrane
Source: Sci Rep. 2016 Sep 15;6:33340. doi: 10.1038/srep33340 (PMC5024136; doi:10.1038/srep33340)
Supplement: Supplementary Information [file srep33340-s1.pdf]

Supplementary information

Oncogenic Mutations Affect Bax Monomer, Dimer, and Pore in Membrane Differentially

Mingzhen Zhang[1], Jie Zheng[1], Ruth Nussinov[2,3*] and Buyong Ma[2*]

[1] Department of Chemical & Biomolecular Engineering, the University of Akron, Akron, Ohio 44325

[2] Basic Science Program, Basic Science Program, Leidos Biomedical Research, Inc. Cancer and Inflammation Program, National Cancer Institute, Frederick, MD 21702, USA

[3] Sackler Inst. of Molecular Medicine, Department of Human Genetics and Molecular Medicine, Sackler School of Medicine, Tel Aviv University, Tel Aviv 69978, Israel

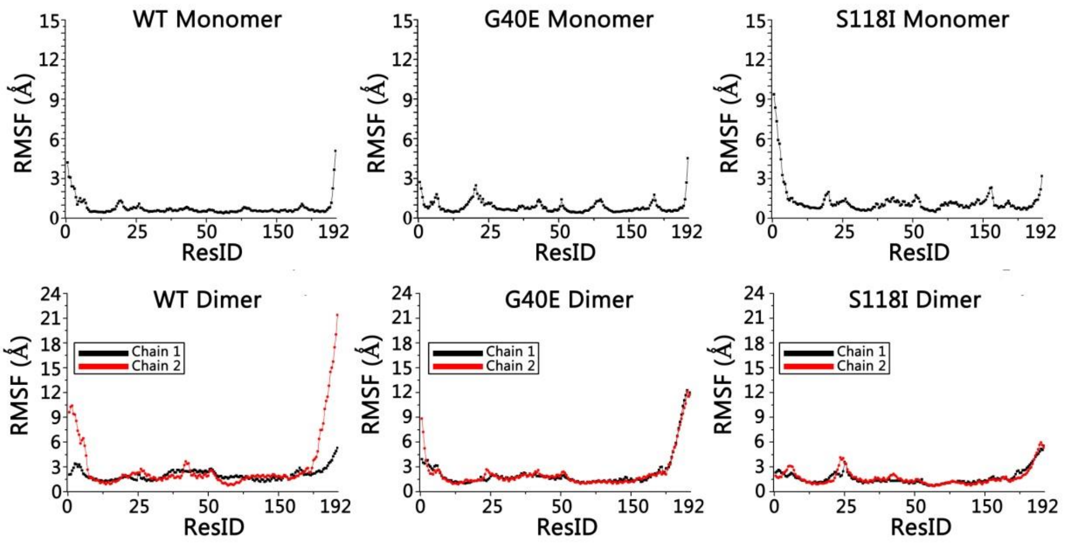

Figure S1. Residue-based backbone RMSF profiles for wild-type, G40E and S118 monomers and dimers. WT denotes the wild type.

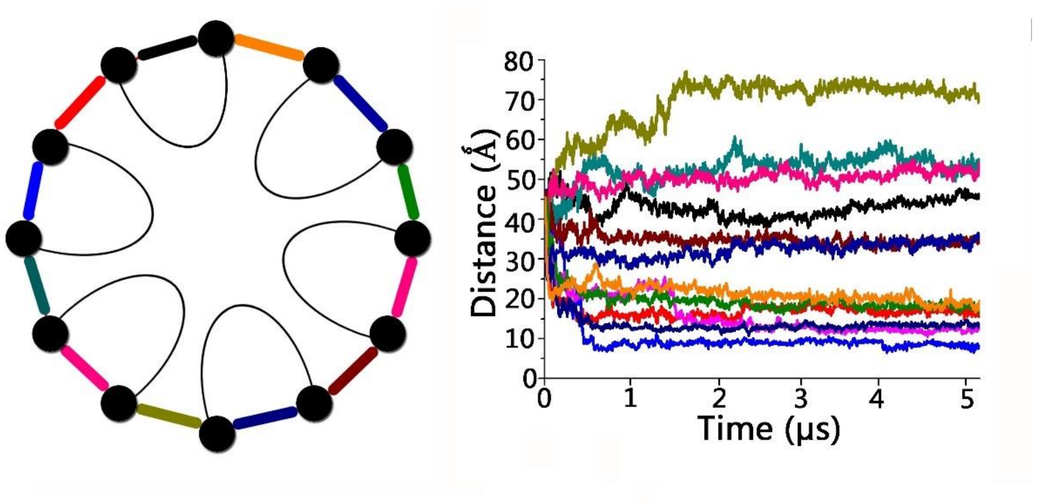

Figure S2. Dimerization of Bax α9 during pore formation simulation. The Bax α9 was initial inserted into membrane without contact, unlike the dimeric conformation in other Bax segments. However, the α9 spontaneously dimerize, increasing overall stability of Bax pore. The plot reports the distances between the center of masses for the different Bax α9 units.

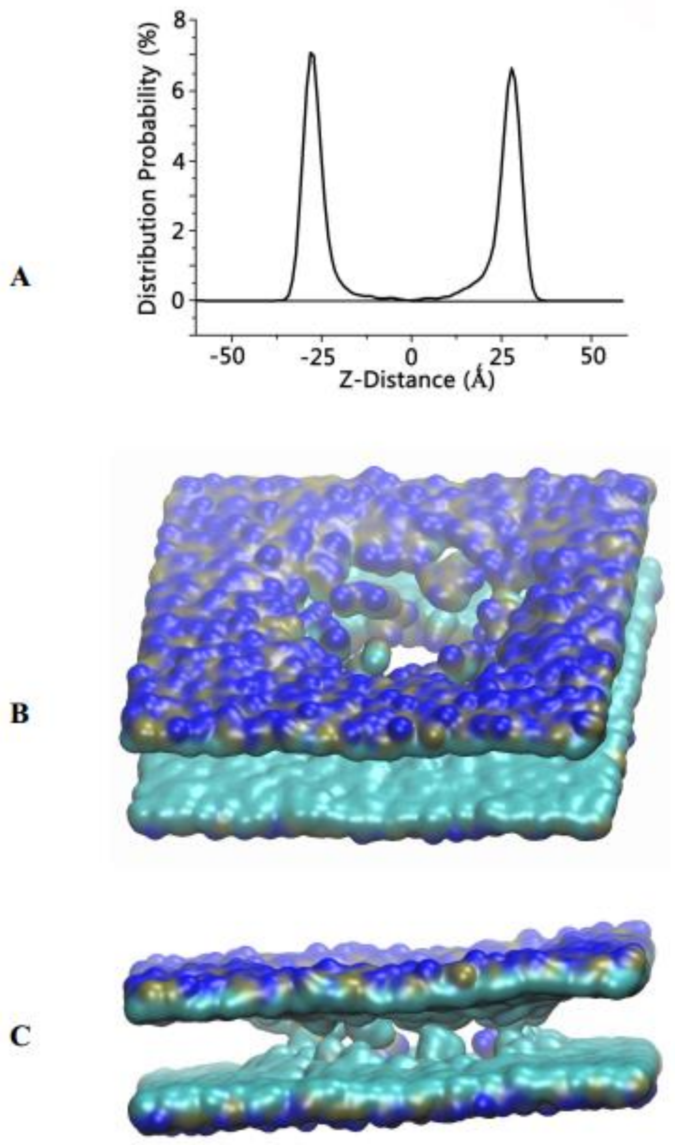

Figure S3. Bax docecamer forms toroidal pore in membrane after 1.5 μs simulation. (A) Distribution of lipid head group perpendicular to the membrane. (B) and (C) Top and side view of lipid head groups of membrane portion of the pore.

Table S1. GMBV energies for the simulated systems.

	$E_{monomer}$ (kcal/mol)	E_{dimer} (kcal/mol)	ΔE (kcal/mol)
Wild Type	-6233.72	-12425.60	41.84
G40E	-6209.00	-12454.00	-36.00
S118I	-6165.67	-12464.40	-133.06
